# Use of the 22C3 anti–PD-L1 antibody to determine PD-L1 expression in multiple automated immunohistochemistry platforms

**DOI:** 10.1371/journal.pone.0183023

**Published:** 2017-08-10

**Authors:** Marius Ilie, Shirin Khambata-Ford, Christiane Copie-Bergman, Lingkang Huang, Jonathan Juco, Veronique Hofman, Paul Hofman

**Affiliations:** 1 Université Côte d'Azur, University Hospital Federation OncoAge, Laboratory of Clinical and Experimental Pathology, Hôpital Pasteur, Nice, France; 2 Université Côte d'Azur, University Hospital Federation OncoAge, Hospital-Related Biobank, Hôpital Pasteur, Nice, France; 3 Université Côte d'Azur, CNRS, INSERM, Institute of Research on Cancer and Ageing of Nice (IRCAN), University Hospital Federation OncoAge, Hôpital Pasteur, Nice, France; 4 Merck & Co., Inc., Kenilworth, New Jersey, United States of America; 5 AP-HP, Groupe Henri Mondor-Albert Chenevier, Departement de Pathologie, Hôpital Henri Mondor, Créteil, France; 6 Université Paris-Est, Faculté de Médecine, Créteil, France; 7 INSERM, Unité U955, Hôpital Henri Mondor, Créteil, France; Universita degli Studi di Napoli Federico II, ITALY

## Abstract

**Background:**

For non–small cell lung cancer (NSCLC), treatment with pembrolizumab is limited to patients with tumours expressing PD-L1 assessed by immunohistochemistry (IHC) using the PD-L1 IHC 22C3 pharmDx (Dako, Inc.) companion diagnostic test, on the Dako Autostainer Link 48 (ASL48) platform. Optimised protocols are urgently needed for use of the 22C3 antibody concentrate to test PD-L1 expression on more widely available IHC autostainers.

**Methods:**

We evaluated PD-L1 expression using the 22C3 antibody concentrate in the three main commercially available autostainers Dako ASL48, BenchMark ULTRA (Ventana Medical Systems, Inc.), and Bond-III (Leica Biosystems) and compared the staining results with the PD-L1 IHC 22C3 pharmDx kit on the Dako ASL48 platform. Several technical conditions for laboratory-developed tests (LDTs) were evaluated in tonsil specimens and a training set of three NSCLC samples. Optimised protocols were then validated in 120 NSCLC specimens.

**Results:**

Optimised protocols were obtained on both the VENTANA BenchMark ULTRA and Dako ASL48 platforms. Significant expression of PD-L1 was obtained on tissue controls with the Leica Bond-III autostainer when high concentrations of the 22C3 antibody were used. It therefore was not tested on the 120 NSCLC specimens. An almost 100% concordance rate for dichotomized tumour proportion score (TPS) results was observed between TPS ratings using the 22C3 antibody concentrate on the Dako ASL48 and VENTANA BenchMark ULTRA platforms relative to the PD-L1 IHC 22C3 pharmDx kit on the Dako ASL48 platform. Interpathologist agreement was high on both LDTs and the PD-L1 IHC 22C3 pharmDx kit on the Dako ASL48 platform.

**Conclusion:**

Availability of standardized protocols for determining PD-L1 expression using the 22C3 antibody concentrate on the widely available Dako ASL48 and VENTANA BenchMark ULTRA IHC platforms will expand the number of laboratories able to determine eligibility of patients with NSCLC for treatment with pembrolizumab in a reliable and concordant manner.

## Introduction

Pembrolizumab is a humanised monoclonal antibody that targets programmed death 1 (PD-1), a negative co-stimulatory receptor expressed on the surface of activated T cells that can be exploited by tumours to evade T-cell–induced anti-tumour activity [1–3]. It has increased clinical activity in tumours that express programmed death ligand 1 (PD-L1) [1,4]. Approximately 23%–28% of patients with advanced non–small cell lung cancer (NSCLC) have a high level of PD-L1 expression, defined as membranous PD-L1 expression on at least 50% of tumour cells, regardless of the staining intensity [1,4].

Pembrolizumab is currently approved as a monotherapy in >60 countries for one or more of the following indications: unresectable or metastatic melanoma; metastatic NSCLC with PD-L1 expression and disease progression on or after platinum-containing chemotherapy; metastatic NSCLC with high PD-L1 expression and no prior systemic chemotherapy; recurrent or metastatic head and neck squamous cell carcinoma with disease progression on or after platinum-containing chemotherapy; adult and paediatric refractory classical non-Hodgkin’s lymphoma; cisplatin-ineligible metastatic urothelial carcinoma; and microsatellite-instability–high solid tumours. It is also approved in combination with pemetrexed and carboplatin as a first-line treatment for patients with metastatic non-squamous NSCLC [5,6]. Currently, monotherapy with pembrolizumab in NSCLC is limited to patients with tumours that express PD-L1 [5,6]. In patients with metastatic NSCLC and progression on or after platinum-containing chemotherapy, pembrolizumab is approved for the treatment of patients with tumour PD-L1 expression ≥1% based on results from the KEYNOTE-010 (NCT01905657) study [4]. In patients with metastatic NSCLC and no prior systemic therapy, pembrolizumab was recently approved in the United States for the treatment of patients with PD-L1 expression ≥50% based on the results of the KEYNOTE-024 (NCT02142738) study [7]. Based on these data, pembrolizumab was approved in conjunction with a companion diagnostic test, the PD-L1 IHC 22C3 pharmDx assay (Dako, Carpinteria, CA) for use on the Dako Autostainer Link 48 (ASL48) platform [8,9]. However, pathology laboratories without the ASL48 platform are currently unable to provide PD-L1 immunohistochemistry (IHC) staining to identify patients with NSCLC suitable for treatment with pembrolizumab. Moreover, while the VENTANA PD-L1 (SP263) assay has been available since May 8, 2017 for the VENTANA BenchMark ULTRA platform in countries in Europe that accept CE marking [10], there is no approved SP263 assay on that platform for pembrolizumab in the vast majority of the world. Furthermore, unlike other antibodies that are designated for research use only (ie, they cannot be used for diagnostic purposes), the 22C3 antibody concentrate is a registered *in-vitro* diagnostic (IVD) antibody, the use of which in combination with laboratory-developed tests is considered to be the most financially sustainable. Thus, there is an urgent need for a reliable protocol that can allow PD-L1 IHC testing using the 22C3 antibody on widely available IHC platforms worldwide.

Our study investigated the 22C3 antibody concentrate (ref: M365329; Dako, Inc.), in the three main commercially available autostainers, Autostainer Link (ASL48; Dako, Inc.), BenchMark ULTRA (Ventana Medical Systems, Inc., Tucson, AZ, USA), and Bond-III (Leica Biosystems, Inc., Buffalo Grove, IL, USA) and compared the results with the ‘gold standard’ PD-L1 IHC 22C3 pharmDx kit (ref: SK006; Dako, Inc.) on the Dako ASL48 platform, in order to develop standardized protocols for PD-L1 IHC using the 22C3 antibody concentrate.

## Methods

### Tumour samples and controls

Archival formalin-fixed paraffin-embedded (FFPE) tumour samples from 120 patients with stage I–IV NSCLC were retrospectively selected from the Laboratory of Clinical and Experimental Pathology and the Nice Hospital-Integrated Biobank (BB-0033-00025) (Hôpital Pasteur, Centre Hospitalier Universitaire de Nice, Nice, France). All tumour specimens were collected, stored, and used with informed written consent from the patients. The study was approved by the local ethics committee (Human Research Ethics Committee, Centre Hospitalier Universitaire de Nice/Tumorothèque BB-0033-00025) and was performed in accordance with the guidelines of the Declaration of Helsinki.

Tumour samples were obtained from patients who underwent surgical resection or biopsy in the departments of pulmonary medicine and thoracic surgery at Hôpital Pasteur between March 2007 and March 2016. Of the 120 tumour specimens evaluated, 95% consisted of surgical resection specimens and the remaining 5% were bronchial biopsies and transbronchial mediastinal lymph node biopsies. A sample was considered eligible for the study if the tumour morphology was preserved and if a minimum of 100 cancer cells were present in the tissue section. Morphologic classification was assigned according to the last World Health Organization/International Association for the Study of Lung Cancer/American Thoracic Society/European Respiratory Society criteria, and confirmed by the immunohistochemical phenotype (thyroid transcription factor-1, p40) by the same three senior lung pathologists who reviewed the NSCLC IHC (MI, VH, and PH). The 120 NSCLC samples were to include 80 adenocarcinomas (60 stage IIIb or IV, 10 stage II, 10 stage I) and 40 squamous cell carcinomas (25 stage IIIb or IV, 10 stage II, 5 stage I). Sources of tumour samples included patients who may have had common genetic alterations such as epidermal growth factor receptor mutations or anaplastic lymphoma kinase translocations.

### Immunohistochemistry

PD-L1 IHC using the PD-L1 IHC 22C3 pharmDx kit on the Dako ASL48 platform was performed according to manufacturer recommendations [10]. The 22C3 antibody in this kit is provided already diluted at an unspecified ratio. For development of high-quality laboratory-developed tests (LDTs), several technical conditions were evaluated (eg, slide thickness 3 μm versus 4 μm, pre-treatment delays, primary antibody dilution, incubation time, amplification systems) using the 22C3 antibody concentrate on the Dako ASL48, VENTANA BenchMark ULTRA and Bond-III platforms (S1 Table). Details of the optimised protocols are described below.

### Optimised Dako ASL48 protocol using the 22C3 concentrate

Specimens were sectioned at a thickness of 3 μm and stained on positively charged glass slides stored at 4°C within 3 days after sectioning. Deparaffinization, rehydration, and antigen retrieval were performed on PT Link (Dako PT100) using the EnVision™ FLEX Target Retrieval Solution, at low pH 6.0 for 53 minutes at room temperature. Following FLEX peroxidase block for 5 minutes, specimens were incubated with primary mouse anti–PD-L1 monoclonal antibody (ref. M365329; Dako, Inc.), using a concentration of 1:50 for 60 minutes at room temperature. Specimens were then incubated with the EnVision™ FLEX+ Mouse LINKER for 30 minutes at room temperature, followed by incubation with the EnVision™ FLEX HRP visualization reagent for 30 minutes at room temperature. The enzymatic conversion of the subsequently added 3,3’-diaminobenzidine tetrahydrochloride (DAB) chromogen for 10 minutes at room temperature followed by DAB enhancer for 5 minutes at room temperature resulted in precipitation of a visible reaction product at the site of antigen. The specimens were then counterstained with haematoxylin and coverslipped. The entire described steps are followed by buffer washes for 5 minutes (EnVision^TM^ FLEX Wash Buffer 20x). Each IHC run contained a positive control (on-slide tonsil tissue) and a negative antibody control (buffer, no primary antibody) (http://dx.doi.org/10.17504/protocols.io.iw9cfh6).

### Optimised VENTANA BenchMark ULTRA protocol

Specimens were sectioned at a thickness of 3 μm and stained on positively charged glass slides stored at 4°C within 3 days after sectioning. Deparaffinization, rehydration, and antigen retrieval were performed by CC1 (prediluted; pH 8.0) antigen retrieval solution (ref. 950–124, Ventana Medical Systems, Inc.), performed on the VENTANA BenchMark ULTRA automated slide stainer for 64 minutes at 100°C. Specimens were incubated with primary mouse anti–PD-L1 monoclonal antibody (ref. M365329; Dako) using a concentration of 1:50 for 32 minutes at 37°C, followed by visualization with the OptiView DAB IHC Detection Kit (Ventana) and OptiView Amplification Kit (Ventana) for 12 minutes. The specimens were then counterstained with haematoxylin II and bluing reagent (Ventana) and coverslipped. Each IHC run contained a positive control (on-slide tonsil tissue) and a negative antibody control (buffer, no primary antibody) (http://dx.doi.org/10.17504/protocols.io.ixacfie).

### Optimised Bond-III Leica protocol

Specimens were sectioned at a thickness of 3 μm and stained on positively charged glass slides stored at 4°C within 3 days after sectioning. Deparaffinization, rehydration, and antigen retrieval were performed by BERS2 (prediluted; pH 9.0) antigen retrieval solution (ref. AR 9640) performed on the Bond-III Leica automated slide stainer for 20 minutes at 100°C. Specimens were incubated with primary mouse anti–PD-L1 monoclonal antibody (ref. M365329; Dako) using a concentration of 1:10 for 2×60 minutes at room temperature, followed by visualisation with the Leica Bond detection kit for 20 minutes at room temperature (Ref. DS 9800). The specimens were then counterstained with haematoxylin and coverslipped. Each IHC run contained a positive control (tonsil tissue)

### Scoring and interpretation of 22C3 PD-L1 staining in 120 NSCLC specimens using Dako ASL48 and VENTANA Benchmark ULTRA platforms and PD-L1 IHC 22C3 pharmDX kit

Tumour proportion score (TPS) was evaluated on samples with at least 100 viable tumour cells in the specimen. TPS was calculated as the percentage of PD-L1–positive cells (defined as complete circumferential or partial cell membrane staining of viable tumour cells with 1+ to 3+ intensity) relative to all viable tumour cells (positive or negative) present in the sample.

Scoring was assessed by three trained pathologists (pathologist A, pathologist B, and pathologist C); pathologists B and C were trained by pathologist A, who underwent a live course specially designed to train pathologists for scoring and interpretation of PD-L1 IHC results that are generated using the PD-L1 IHC 22C3 pharmDx kit (Dako) on the Dako ASL48 platform. Each pathologist scored the same stained glass slides independently and was masked as to the staining protocol used on a particular slide. All slides generated from staining on one platform (with no knowledge of which platform) were read at the same time. Slides stained from each platform were read independently at different times. Moreover, between each analysis for a given platform the order of samples was randomly rearranged by the technicians.

Micrographs from the slides used for assay development (ie, tonsil and three lung cancer specimens) were created by whole-slide scanning, using a Nanozoomer HT 2.0 Scanner (Hamamatsu Photonics, Japan). Automated analysis was used to complement the manual analysis made by pathologists, allowing differences between tests, such as variation in intensity and number of cells for the development of LDTs to be seen. While intensity is not used in determining the TPS, the automatic analysis may provide clues on small variations, approximately 1%, which is an important score in certain clinical scenarios, especially in second-line treatment.

To complement the IHC manual scoring, automated densitometry measurements of immunoprecipitates in scanned whole slides were made by using the Interactive Learning and Segmentation Toolkit that allows for tissue recognition within the Calopix software (Tribvn, Châtillon, France) in order to analyse only the epithelial component (membranous/cytoplasmic). The Calopix IHC algorithm was then applied to each scanned whole-slide.

### Statistical analysis

TPS scores using optimised protocols for two LDTs using the 22C3 antibody concentrate on the Dako ASL48 and VENTANA BenchMark ULTRA platforms were compared with the PD-L1 IHC 22C3 pharmDx kit on the Dako ASL48 platform (gold standard). The sample size of 120 NSCLC samples for evaluating the concordance of LDTs using the 22C3 antibody and the PD-L1 IHC 22C3 pharmDx kit was based on precision of estimates of positive and negative percentage agreement to ensure that the margin of error was near or below 5%.

Statistical analyses assessing agreement were performed on TPS scores as continuous variables as well as on dichotomized variables using two positivity cut points: TPS positive (≥1%) and TPS strongly positive (≥50%). Positive percentage agreement (PPA) and negative percentage agreement (NPA) were calculated for the comparison between the dichotomized TPS results using each of the 22C3 concentrate LDTs relative to the TPS results observed with the PD-L1 IHC 22C3 pharmDx kit on the Dako ASL48 platform. 95% confidence intervals were calculated using 2-sided Wilson score confidence limits. Intraclass correlation coefficients were calculated for the comparison of TPS raw scores as continuous variables from each of the 22C3 concentrate LDTs, relative to the TPS score observed with the 22C3 pharmDx kit. κ scores were calculated for dichotomized TPS results at the 1% or 50% level to assess the interpathologist agreement for each of the LDTs and for the PD-L1 IHC 22C3 pharmDx kit.

## Results

### Development of the LDTs

**Fig 1:**
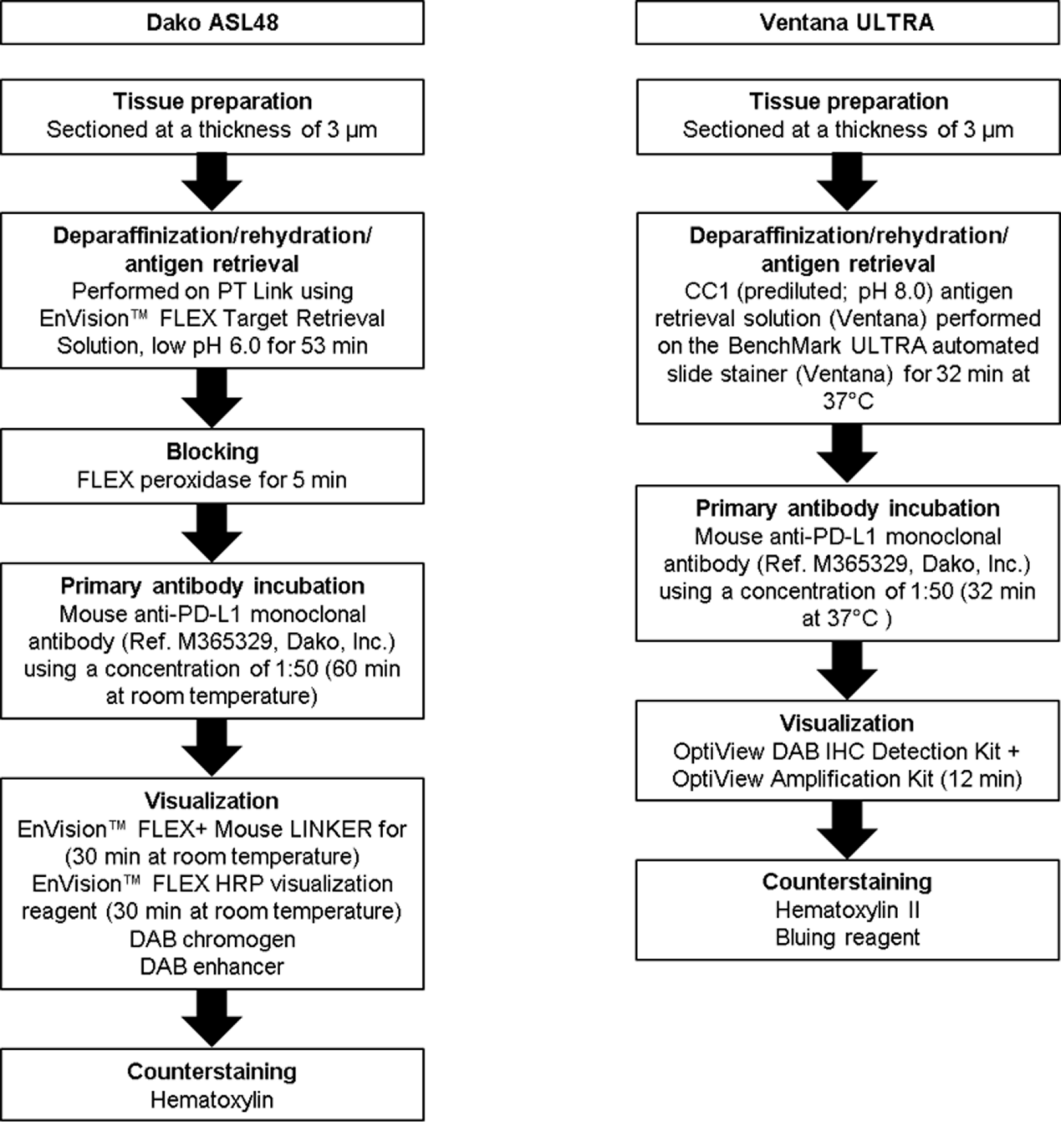
Optimised protocols for PD-L1 IHC assays using the 22C3 antibody concentrate on the Dako ASL48 and VENTANA BenchMark ULTRA platforms. PD-L1, programmed death ligand 1; IHC, immunohistochemistry; ASL48, Autostainer Link 48; DAB, 3,3’-diaminobenzidine tetrahydrochloride.

**Fig 2:**
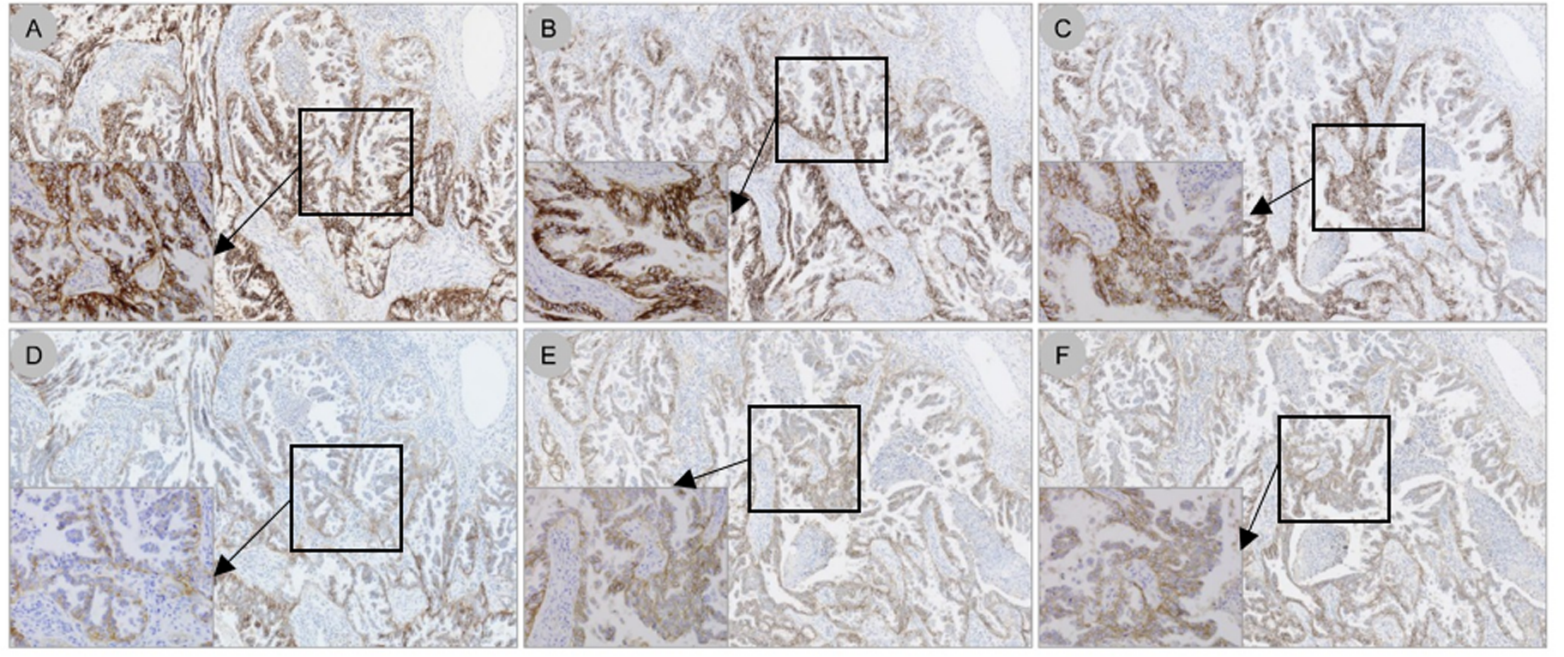
PD-L1 staining patterns on NSCLC specimens using the 22C3 antibody concentrate on the Dako ASL48 platform (LDT) compared with the PD-L1 IHC 22C3 pharmDx kit on the Dako ASL48 platform (gold standard). (*A*) The PD-L1 22C3 pharmDx kit on the Dako ASL48 platform; (*B*) optimised LDT (primary antibody dilution 1:50, 30-minute incubation); (*C*) LDT using primary antibody dilution 1:100, 30-minute incubation; (*D*) primary antibody dilution 1:200, 30-minute incubation; (*E*) primary antibody dilution 1:100, 60-minute incubation; (*F*) primary antibody dilution 1:100, 120-minute incubation. Original magnification 5×. Inserts, original magnification 40×. PD-L1, programmed death ligand 1; NSCLC, non–small cell lung cancer; ASL48, Autostainer Link 48; LDT, laboratory-developed test; IHC, immunohistochemistry.

A total of 10 protocols were evaluated on the Dako ASL48, 15 protocols on the VENTANA BenchMark ULTRA, and 11 protocols on the Bond-III platforms in serial sections from a tonsil specimen (see S1 Table; S1 Fig, S2 Fig, S3 Fig and S4 Fig). Based on comparison with the PD-L1 IHC 22C3 pharmDx on the Dako ASL48 platform, both by manual and automated scoring, some conditions were selected for further development in a training set of three NSCLC resection specimens with TPS scores of 70%, 90%, and 100% (determined using the PD-L1 IHC 22C3 pharmDx kit on the Dako ASL48 platform) and are detailed in the Methods section. The Bond-III platform required high levels of 22C3 antibody concentrate (2 incubations of 60 minutes at 1:10 dilution) to reach the gold standard of PD-L1 IHC 22C3 pharmDx on the Dako ASL48 platform on tonsil and the three NSCLC samples (see S5 Fig). These suboptimal technical conditions were considered to be financially unfeasible for further testing of all 120 NSCLC specimens. Therefore, the validation on the 120 NSCLC specimens using 22C3 antibody concentrate was performed only with Dako ASL48 and VENTANA BenchMark ULTRA platforms (Fig 1). Staining patterns on NSCLC specimens using the 22C3 antibody concentrate on the Dako ASL48 platform, compared with the PD-L1 IHC 22C3 pharmDx kit on the Dako ASL48 platform (gold standard), are shown in Fig 2. Using a TPS cut point of ≥1%, 54/120 cases (45%) were deemed PD-L1 positive, while using a TPS cut point of ≥50%, 29/120 (24%) were considered positive for PD-L1.

**Fig 3:**
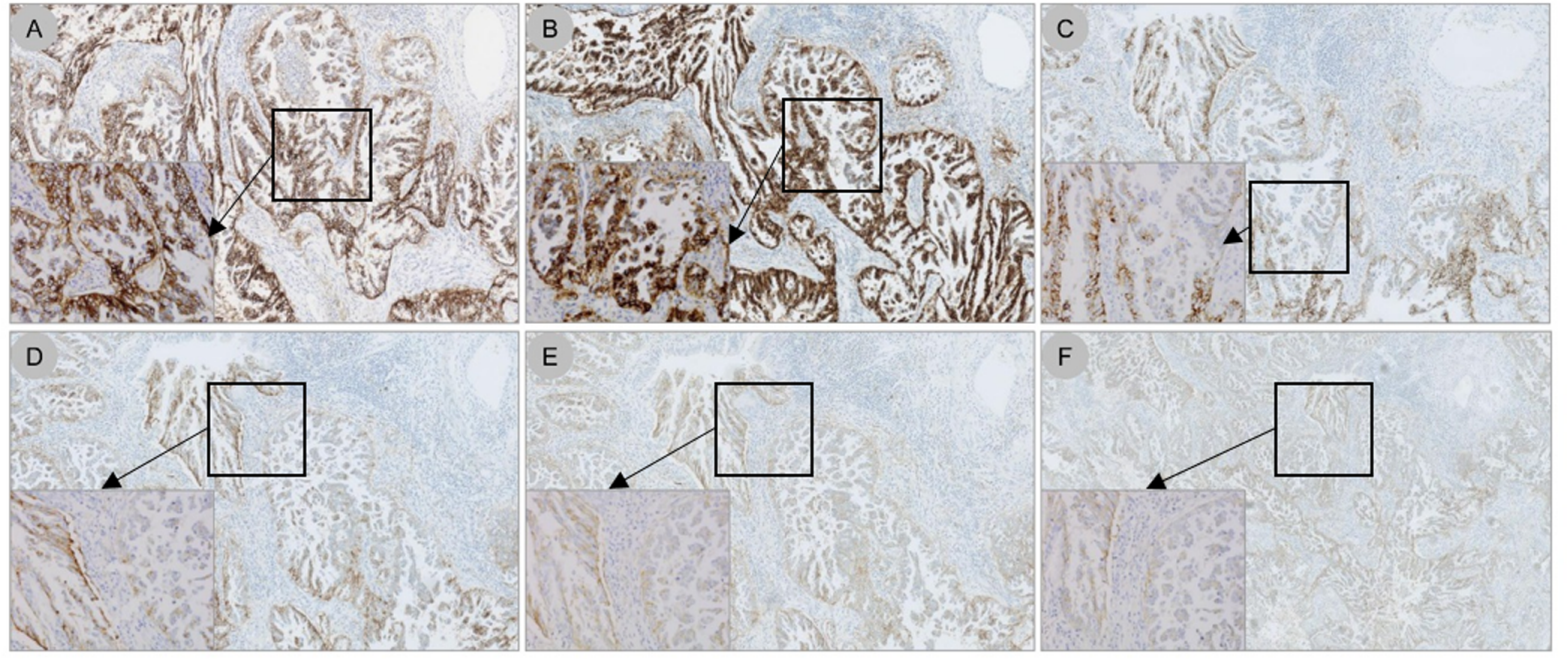
PD-L1 staining patterns on NSCLC specimens using the 22C3 antibody concentrate on the VENTANA BenchMark ULTRA platform (LDT) compared with the PD-L1 IHC 22C3 pharmDx kit on the Dako ASL48 platform (gold standard). (*A*) The PD-L1 IHC 22C3 pharmDx kit on the Dako ASL48 platform; (*B*) optimised LDT (CC1 64 minutes, 22C3 primary antibody dilution 1:50, OptiView amplification 12 minutes); (*C*) CC1 32 minutes, primary antibody dilution 1:50, OptiView amplification 12 minutes; (*D*) CC1 64 minutes, primary antibody dilution 1:50, OptiView amplification 4 minutes; (*E*) CC1 32 minutes, primary antibody dilution 1:100, OptiView amplification 12 minutes; (*F*) CC1 64 minutes, primary antibody dilution 1:100, OptiView amplification 12 minutes. Original magnification 5×. Inserts, original magnification 40×. PD-L1, programmed death ligand 1; NSCLC, non–small cell lung cancer; ASL48, Autostainer Link 48; LDT, laboratory-developed test; IHC, immunohistochemistry.

The optimised protocol for the PD-L1 IHC assay using the 22C3 antibody concentrate on the VENTANA BenchMark ULTRA platform is described in the Methods section (Fig 1). Staining patterns on NSCLC specimens using the 22C3 antibody concentrate on the VENTANA BenchMark ULTRA platform, compared with the PD-L1 IHC 22C3 pharmDx kit on the Dako ASL48 platform (gold standard), are shown in Fig 3. Using a TPS ≥1%, 54/120 cases (45%) were considered positive for PD-L1, while using a TPS ≥50%, 29/120 cases (24%) were PD-L1 positive.

### Agreement evaluation of each platform versus gold standard

For each of the three pathologists, a 100% concordance rate for dichotomized TPS results at <1% versus ≥1% was observed between TPS ratings from each of the 22C3 antibody concentrate LDTs relative to the gold standard observed with the PD-L1 IHC 22C3 pharmDx kit on the Dako ASL48 platform (Table 1). Similarly, a 100% concordance rate for dichotomized TPS results at <50% versus ≥50% was observed between TPS ratings from each of the two 22C3 antibody concentrate LDTs relative to the gold standard, except that pathologist C evaluated 29 out of 30 samples as TPS ≥50% for the VENTANA BenchMark ULTRA versus the gold standard. The intraclass correlation coefficients, used to measure the correlation of TPS score as continuous variable, were between 98.7% and 99.9% (Table 2). Although raw TPS scores differed slightly between each of the 22C3 concentrate LDTs and the gold standard, the assigned TPS categories from each of the 22C3 antibody concentrate LDTs were almost the same as the assigned TPS categories from the gold standard except for one observation (see S6 Fig).

**Table 1:** PPA and NPA for each of the PD-L1 IHC assays using the 22C3 antibody concentrate on the Dako ASL48 and VENTANA BenchMark ULTRA platforms (LDTs) relative to the PD-L1 IHC 22C3 pharmDx kit on the Dako ASL48 platform (gold standard).

LDT	TPS cut point	Pathologist	pharmDx NPA	pharmDx PPA	Total agreement
22C3 on Dako ASL48	≥1%	A	66/66 (100%)	54/54 (100%)	120/120 (100%)
		B	66/66 (100%)	54/54 (100%)	120/120 (100%)
		C	66/66 (100%)	54/54 (100%)	120/120 (100%)
22C3 on VENTANA BenchMark ULTRA	≥1%	A	66/66 (100%)	54/54 (100%)	120/120 (100%)
		B	66/66 (100%)	54/54 (100%)	120/120 (100%)
		C	66/66 (100%)	54/54 (100%)	120/120 (100%)
22C3 on Dako ASL48	≥50%	A	91/91 (100%)	29/29 (100%)	120/120 (100%)
		B	91/91 (100%)	29/29 (100%)	120/120 (100%)
		C	90/90 (100%)	30/30 (100%)	120/120 (100%)
22C3 on VENTANA BenchMark ULTRA	≥50%	A	91/91 (100%)	29/29 (100%)	120/120 (100%)
		B	91/91 (100%)	29/29 (100%)	120/120 (100%)
		C	90/90 (100%)	29/30 (97%)	119/120 (99%)

PPA, positive percentage agreement; NPA, negative percentage agreement; IHC, immunohistochemistry; LDT, laboratory-developed test; ASL48, Autostainer Link 48; PD-L1, programmed death ligand 1; TPS, tumour proportion score.

**Table 2:** Intraclass correlation coefficient of TPS rating for each of the PD-L1 IHC assays using the 22C3 antibody concentrate on the Dako ASL48 and VENTANA BenchMark ULTRA platforms (LDTs) relative to the PD-L1 IHC 22C3 pharmDx kit on the Dako ASL48 platform (gold standard).

LDT	Pathologist	Intraclass Correlation Coefficient
22C3 on Dako ASL48	A	99.3%
	B	99.3%
	C	98.7%
22C3 on VENTANA BenchMark ULTRA	A	99.9%
	B	99.7%
	C	99.4%

TPS, tumour proportion score; PD-L1, programmed death ligand 1; IHC, immunohistochemistry; ASL48, Autostainer Link 48; LDT, laboratory-developed test.

Interpathologist agreement was high on both LDTs and the PD-L1 IHC 22C3 pharmDx kit on the Dako ASL48 platform (gold standard). Using a TPS cut point of ≥1%, κ scores for interpathologist agreement were 1 for both LDTs and the gold standard. Using a TPS cut point of ≥50%, κ scores were 1 for interpathologist agreement for VENTANA BenchMark ULTRA, and 0.99 for the gold standard and Dako ASL48.

## Discussion

The PD-L1 IHC 22C3 pharmDx kit was recently approved by the US Food and Drug Administration as a companion diagnostic assay to identify patients with metastatic NSCLC who are most likely to respond to pembrolizumab [8,9]. The PD-L1 IHC 22C3 pharmDx kit is a qualitative IHC assay using monoclonal mouse anti–PD-L1 antibody clone 22C3 intended for use in the detection of PD-L1 protein in FFPE NSCLC tissue specimens using the EnVision FLEX visualization system on the Dako ASL48 platform [10]. The recent release of the 22C3 antibody concentrate by Dako would enable accurate PD-L1 testing on other widely available IHC platforms worldwide.

We have developed optimised protocols for determining PD-L1 expression by IHC using the 22C3 antibody concentrate (ref. M365329; Dako, Inc.) on both the VENTANA BenchMark ULTRA and Dako ASL48 platforms. The 22C3 antibody concentrate may be compatible with the Leica Bond-III autostainer provided that high concentration (1:10) is used. In addition, send-out testing may be considered on national validated and accredited platforms for those laboratories that carry a Leica Bond III. TPS ratings demonstrated high concordance compared with the gold standard 22C3 pharmDx kit on the Dako ASL48 for both LDTs.

In our protocol development experiments, we first used tonsil specimens as widely recommended positive controls, with the highest expression of PD-L1 in crypt epithelium, macrophages homing into the germinal centres, and interfollicular mononuclear leucocytes. However, when several protocols at 1:50 dilution, different by only subtle modifications of the technical conditions (ie, CC1 32 versus 64 min, amplification 4 versus 12 min; see S1 Table) were further tested on a training set of three NSCLC samples, some degree of variability of the staining (ie, faint intensity, incomplete membranous expression) was observed when compared with the gold standard PD-L1 IHC 22C3 pharmDx kit. As these variations were troublesome to interpret by manual interpretation only, the analysis was supported by automated analysis of the digitized whole slides (see S2 Fig, S4 Fig, S7 Fig and S8 Fig). To select the most accurate LDT in comparison to the gold standard 22C3 pharmDx kit, the automated analysis captured those subtle differences that were more difficult to analyse by manual interpretation only. Given the complexity of the transfer of such analysis in routine practice, we did not use the automated analysis on all 120 NSCLC tumour samples. While antigenic heterogeneity on serial sections of tonsil specimens may be limited, the spatial distribution of heterogeneity of PD-L1 expression may be more frequent in serial sections of NSCLC FFPE specimens [11–13].

Thus, we recommend that a highly PD-L1–positive (≥50% positive tumour cells) NSCLC sample should be used instead to provide a dynamic range of PD-L1 staining for intensity and spatial distribution. The ideal control would be weakly positive staining NSCLC tissue, as this would provide greater sensitivity for detecting degradation of reagents as well as intratumour spatial heterogeneity. Additionally, it is important to note that pathologist training is necessary for correct interpretation of PD-L1 staining on IHC [14,15]. In this study, staining was interpreted by three trained pathologists, resulting in excellent interpathologist agreement on TPS ratings with κ scores of 0.99–1 across platforms.

Our study has a number of limitations, including its retrospective nature, the fact that it was conducted at a single institution, and the fact that no patient was treated with pembrolizumab. To overcome these limitations, national efforts are currently ongoing to validate LDTs in conjunction with pembrolizumab treatment in prospective clinical trials (ie, French National AcSé Program).

To date, only one study has investigated whether it is possible to adapt Dako’s PD-L1 IHC 22C3 pharmDx assay to the BenchMark XT platform (Ventana) [16]. However, using only the prediluted antibody without all the other reagents provided with the pharmDx kit may not be practical [17].

Another harmonization effort for PD-L1 testing was recently made by the Nordic immunohistochemical Quality Control organization (NordiQC, Aalborg University Hospital, Aalborg, Denmark; see http://www.nordiqc.org/downloads/assessments/96_102.pdf) [18]. While our LDTs were similar to those developed by NordiQC, there were minor differences with the assessment of accuracy of the PD-L1 LDTs. The NordiQC used tissue microarray (TMA) spots from cell lines, placenta, and tonsil samples, and from 75 cases of NSCLC. Considering the high intratumoural heterogeneity of PD-L1 expression, this may not be a preferred way to assess such accuracy [13,14]. As noted above, the spatial distribution of heterogeneity of PD-L1 expression is frequent in serial sections of NSCLC FFPE specimens. In our study, we used whole tumour sections from 120 patients with NSCLC.

Availability of standardised protocols for determining PD-L1 expression on the widely available Dako ASL48 and VENTANA BenchMark ULTRA IHC platforms will expand the number of laboratories able to determine eligibility of patients with NSCLC for treatment with pembrolizumab in a reliable and concordant manner. Moreover, using the 22C3 antibody concentrate with the specific reagents for each IHC platform provides greater practicality for pathology laboratories worldwide. Development of protocols for PD-L1 IHC using the 22C3 antibody concentrate on other commercially available IHC platforms such as VENTANA BenchMark XT and Dako Omnis should also be urgently considered.

## Supporting information

S1 Table Technical conditions evaluated for each LDT. Shaded rows represent conditions used in the optimised protocols. LDT, laboratory-developed test; PD-L1, programmed death ligand 1; IHC, immunohistochemistry; ASL48, Autostainer Link 48; na, not applicable.
(DOCX)

S1 Fig PD-L1 staining patterns on tonsil specimen using the 22C3 antibody concentrate on the Dako ASL48 platform compared with the PD-L1 IHC 22C3 pharmDx kit on the Dako ASL48 platform (gold standard). Technical conditions used are indicated. Original magnification 5×. ×. PD-L1, programmed death ligand 1; IHC, immunohistochemistry; ASL48, Autostainer Link 48; LDT, laboratory-developed test; Ab, antibody.
(TIF)

S2 Fig Automated immunohistochemistry quantification of the digital images using Calopix software of cases presented in S1 Fig (blue = negative, yellow = low intensity, orange = moderate intensity, and red = high intensity). (TIF)

S3 Fig PD-L1 staining patterns on tonsil specimen using the 22C3 antibody concentrate on the VENTANA BenchMark ULTRA platform compared with the PD-L1 IHC 22C3 pharmDx kit on the Dako ASL48 platform (gold standard). Technical conditions used are indicated. Original magnification 5×. PD-L1, programmed death ligand 1; IHC, immunohistochemistry; ASL48, Autostainer Link 48; LDT, laboratory-developed test; Ab, antibody.
(TIF)

S4 Fig Automated immunohistochemistry quantification of the digital images using Calopix software of cases presented in S3 Fig (blue = negative, yellow = low intensity, orange = moderate intensity, and red = high intensity). (TIF)

S5 Fig PD-L1 staining patterns on tonsil specimen using the 22C3 antibody concentrate on the Leica Bond-III platform compared with the PD-L1 IHC 22C3 pharmDx kit on the Dako ASL48 platform (gold standard). Technical conditions used are indicated. Original magnification 5×. PD-L1, programmed death ligand 1; IHC, immunohistochemistry; ASL48, Autostainer Link 48; LDT, laboratory-developed test; Ab, antibody.
(TIF)

S6 Fig **Pathologist ratings of TPS on each of the PD-L1 IHC assays using the 22C3 antibody concentrate on the Dako ASL48 (*A-C*) and VENTANA BenchMark ULTRA platforms (*D-F*) (LDTs) relative to the PD-L1 IHC 22C3 pharmDx kit on the Dako ASL48 platform (gold standard).** Note that the number within the points are number of samples. TPS, tumour proportion score; PD-L1, programmed death ligand 1; IHC, immunohistochemistry; ASL48, Autostainer Link 48; LDT, laboratory-developed test; Ab, antibody.
(TIF)

S7 Fig Automated immunohistochemistry quantification of the digital images using Calopix software of cases presented in Fig 2 (blue = negative, yellow = low intensity, orange = moderate intensity, and red = high intensity). (TIF)

S8 Fig Automated immunohistochemistry quantification of the digital images using Calopix software of cases presented in Fig 3 (blue = negative, yellow = low intensity, orange = moderate intensity, and red = high intensity). (TIF)
