# Correction: Use of the 22C3 anti-PD-L1 antibody to determine PD-L1 expression in multiple automated immunohistochemistry platforms

**DOI:** 10.1371/journal.pone.0186537

**Published:** 2017-10-13

**Authors:** Marius Ilie, Shirin Khambata-Ford, Christiane Copie-Bergman, Lingkang Huang, Jonathan Juco, Veronique Hofman, Paul Hofman

Fig 1 is incorrect. The authors have provided a corrected version here.

**Fig 1:**
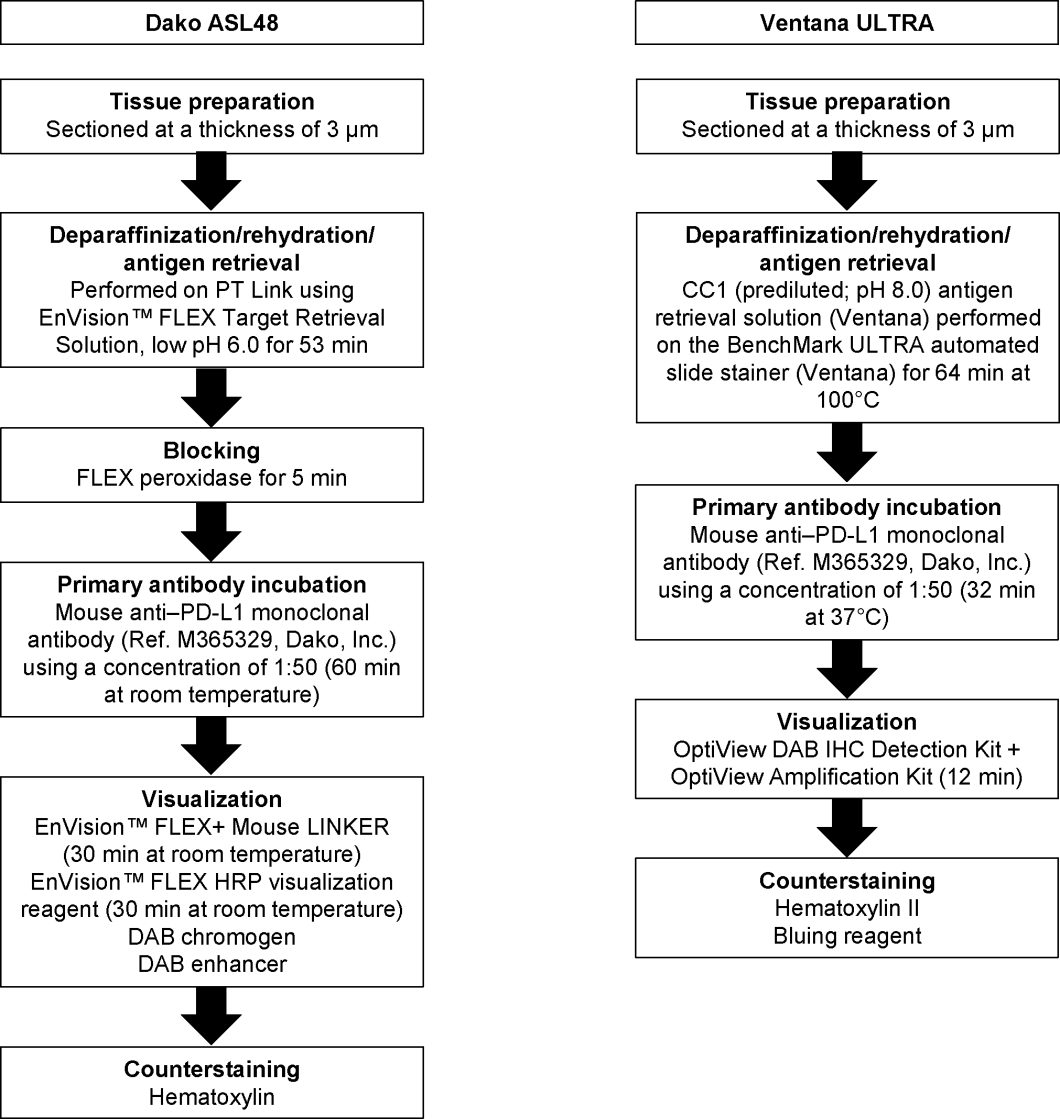
Optimised protocols for PD-L1 IHC assays using the 22C3 antibody concentrate on the Dako ASL48 and VENTANA BenchMark ULTRA platforms. PD-L1, programmed death ligand 1; IHC, immunohistochemistry; ASL48, Autostainer Link 48; DAB, 3,3’-diaminobenzidine tetrahydrochloride.
