# Supplementary material for: Use of the 22C3 anti–PD-L1 antibody to determine PD-L1 expression in multiple automated immunohistochemistry platforms
Source: PLoS One. 2017 Aug 10;12(8):e0183023. doi: 10.1371/journal.pone.0183023 (PMC5552229; doi:10.1371/journal.pone.0183023)
Supplement: S1 Table — Shaded rows represent conditions used in the optimised protocols. LDT, laboratory-developed test; PD-L1, programmed death ligand 1; IHC, immunohistochemistry; ASL48, Autostainer Link 48; na, not applicable. (DOCX) [file pone.0183023.s001.docx]

**S1Table**

| **PD-L1 IHC 22C3 pharmDx kit on the Dako ASL48 platform** | | | | | | | |
| --- | --- | --- | --- | --- | --- | --- | --- |
| **Pre-treatment** | **Pre-treatment  (min)** | **Primary antibody  dilution** | **Primary antibody incubation  (min)** | **Primary antibody temperature (°C)** | **Detection kit** | **Amplification  (min)** | **Thickness** |
| **LOW PH6** | **53** | **Prediluted** | **30** | **Ambient** | **pharmDx** | **na** | **4 µm** |
| LOW PH6 | 53 | Prediluted | 30 | Ambient | pharmDx | na | 3 µm |
| **22C3 antibody concentrate on the Dako ASL48 platform** | | | | | | | |
| **Pre-treatment** | **Pre-treatment  (min)** | **Primary antibody dilution** | **Primary antibody incubation  (min)** | **Primary antibody temperature (°C)** | **Detection kit** | **Amplification  (min)** | **Thickness** |
| LOW PH6 | 53 | 1:50 | 30 | Ambient | FLEX | na | 4 µm |
| LOW PH6 | 53 | 1:100 | 30 | Ambient | FLEX | na | 4 µm |
| LOW PH6 | 53 | 1:200 | 30 | Ambient | FLEX | na | 4 µm |
| LOW PH6 | 53 | 1:50 | 30 | Ambient | FLEX | na | 3 µm |
| **LOW PH6** | **53** | **1:50** | **60** | **Ambient** | **FLEX** | **na** | **3 µm** |
| LOW PH6 | 53 | 1:100 | 30 | Ambient | FLEX | na | 3 µm |
| LOW PH6 | 53 | 1:100 | 60 | Ambient | FLEX | na | 3 µm |
| LOW PH6 | 53 | 1:100 | 120 | Ambient | FLEX | na | 3 µm |
| LOW PH6 | 53 | 1:200 | 30 | Ambient | FLEX | na | 3 µm |
| **22C3 antibody concentrate on the Leica BOND-III platform** | | | | | | | |
| **Pre-treatment** | **Pre-treatment  (min)** | **Primary antibody dilution** | **Primary antibody incubation  (min)** | **Primary antibody temperature (°C)** | **Detection kit** | **Amplification  (min)** | **Thickness** |
| BERS1 | 20 | 1:25 | 20 | Ambient | LEICA Bond | na | 3 µm |
| BERS1 | 20 | 1:50 | 20 | Ambient | LEICA Bond | na | 3 µm |
| BERS1 | 20 | 1:100 | 20 | Ambient | LEICA Bond | na | 3 µm |
| BERS2 | 20 | 1:25 | 20 | Ambient | LEICA Bond | na | 3 µm |
| BERS2 | 20 | 1:50 | 20 | Ambient | LEICA Bond | na | 3 µm |
| BERS2 | 20 | 1:100 | 20 | Ambient | LEICA Bond | na | 3 µm |
| BERS1 | 20 | 1:50 | 20 | Ambient | LEICA Bond | yes | 3 µm |
| BERS2 | 20 | 1:100 | 20 | Ambient | LEICA Bond | yes | 3 µm |
| BERS1 | 20 | 1:25 | 60x2 | Ambient | LEICA Bond | na | 3 µm |
| BERS2 | 20 | 1:25 | 60x2 | Ambient | LEICA Bond | na | 3 µm |
| BERS2 | 20 | 1:10 | 60x2 | Ambient | LEICA Bond | na | 3 µm |
| **22C3 antibody concentrate on the Ventana BenchMark ULTRA platform** | | | | | | | |
| **Pre-treatment** | **Pre-treatment  (min)** | **Primary antibody  dilution** | **Primary antibody incubation  (min)** | **Primary antibody temperature (°C)** | **Detection kit** | **Amplification  (min)** | **Thickness** |
| CC1 | 64 | 1:50 | 32 | 37 | OptiView | 4 | 4 µm |
| CC1 | 64 | 1:50 | 32 | 37 | OptiView | 12 | 4 µm |
| **CC1** | **64** | **1:50** | **32** | **37** | **OptiView** | **12** | **3 µm** |
| CC1 | 64 | 1:50 | 32 | 37 | OptiView | 4 | 3 µm |
| CC1 | 32 | 1:50 | 32 | 37 | OptiView | 12 | 3 µm |
| CC1 | 64 | 1:50 | 32 | 37 | OptiView | 4 | 3 µm |
| CC1 | 64 | 1:50 | 32 | 37 | OptiView | 12 | 3 µm |
| CC1 | 32 | 1:100 | 32 | 37 | OptiView | 12 | 3 µm |
| CC1 | 64 | 1:100 | 32 | 37 | OptiView | 12 | 3 µm |
| CC1 | 32 | 1:100 | 32 | 37 | OptiView | 4 | 3 µm |
| CC1 | 64 | 1:100 | 32 | 37 | ultraView | 8 | 3 µm |
| CC1 | 64 | 1:100 | 60 | 37 | ultraView | 8 | 3 µm |
| CC1 | 64 | 1:100 | 120 | 37 | ultraView | 8 | 3 µm |
