# Supplementary figures and images for: Use of the 22C3 anti–PD-L1 antibody to determine PD-L1 expression in multiple automated immunohistochemistry platforms
Source: PLoS One. 2017 Aug 10;12(8):e0183023. doi: 10.1371/journal.pone.0183023 (PMC5552229; doi:10.1371/journal.pone.0183023)

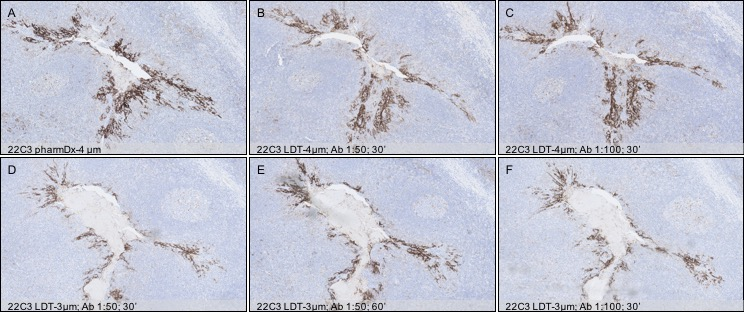

Supplement: S1 Fig — Technical conditions used are indicated. Original magnification 5×. ×. PD-L1, programmed death ligand 1; IHC, immunohistochemistry; ASL48, Autostainer Link 48; LDT, laboratory-developed test; Ab, antibody. (TIF) [file pone.0183023.s002.tif]

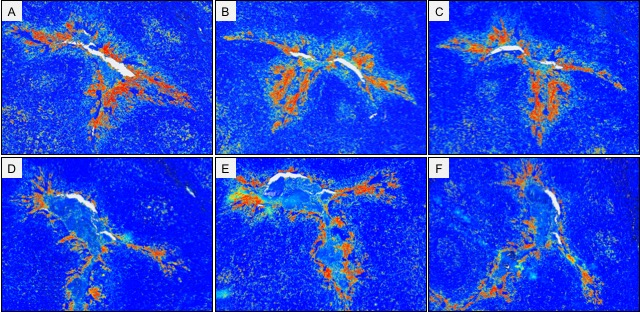

Supplement: S2 Fig — (TIF) [file pone.0183023.s003.tif]

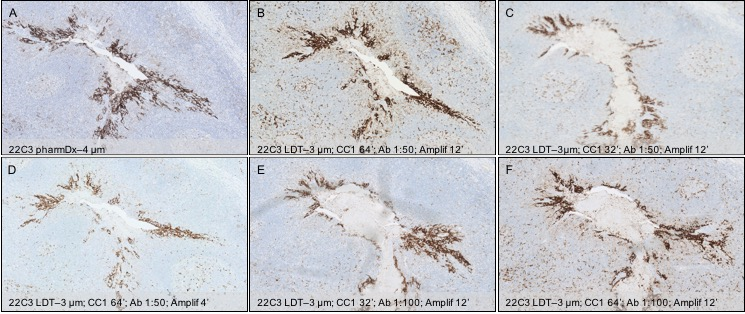

Supplement: S3 Fig — Technical conditions used are indicated. Original magnification 5×. PD-L1, programmed death ligand 1; IHC, immunohistochemistry; ASL48, Autostainer Link 48; LDT, laboratory-developed test; Ab, antibody. (TIF) [file pone.0183023.s004.tif]

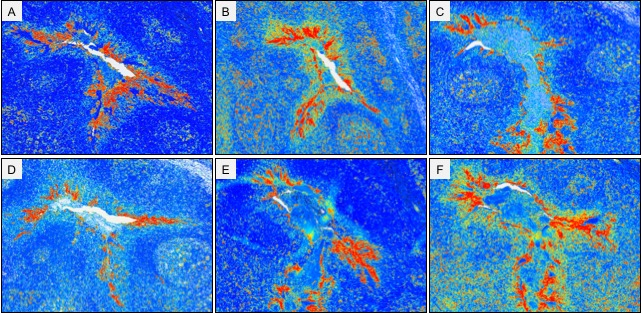

Supplement: S4 Fig — (TIF) [file pone.0183023.s005.tif]

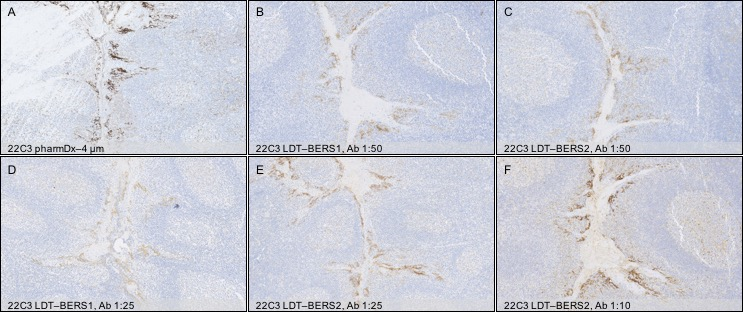

Supplement: S5 Fig — Technical conditions used are indicated. Original magnification 5×. PD-L1, programmed death ligand 1; IHC, immunohistochemistry; ASL48, Autostainer Link 48; LDT, laboratory-developed test; Ab, antibody. (TIF) [file pone.0183023.s006.tif]

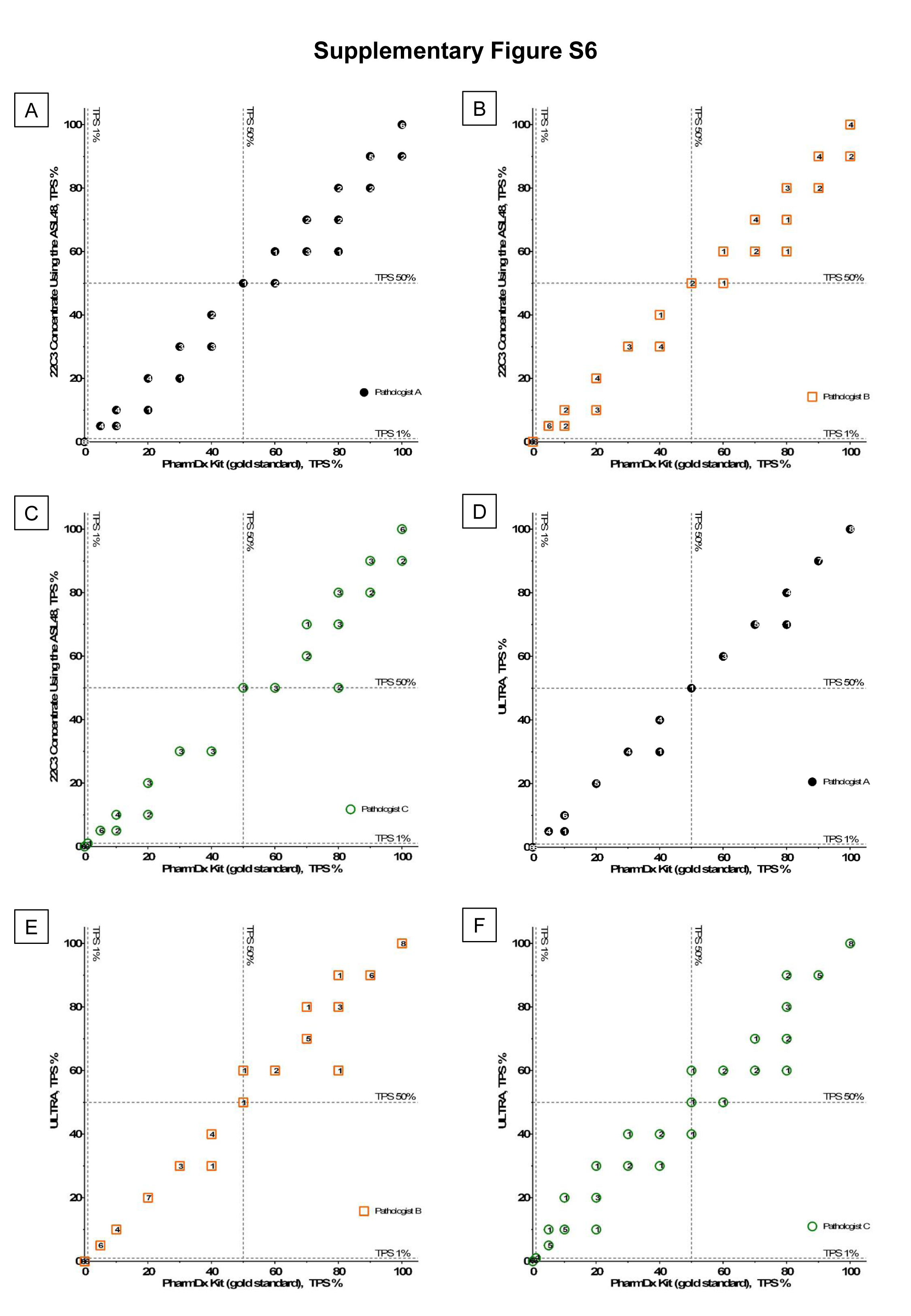

Supplement: S6 Fig — Pathologist ratings of TPS on each of the PD-L1 IHC assays using the 22C3 antibody concentrate on the Dako ASL48 (A-C) and VENTANA BenchMark ULTRA platforms (D-F) (LDTs) relative to the PD-L1 IHC 22C3 pharmDx kit on the Dako ASL48 platform (gold standard). Note that the number within the points are number of samples. TPS, tumour proportion score; PD-L1, programmed death ligand 1; IHC, immunohistochemistry; ASL48, Autostainer Link 48; LDT, laboratory-developed test; Ab, antibody. (TIF) [file pone.0183023.s007.tif]

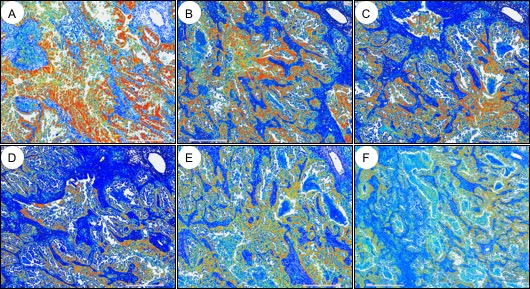

Supplement: S7 Fig — (TIF) [file pone.0183023.s008.tif]

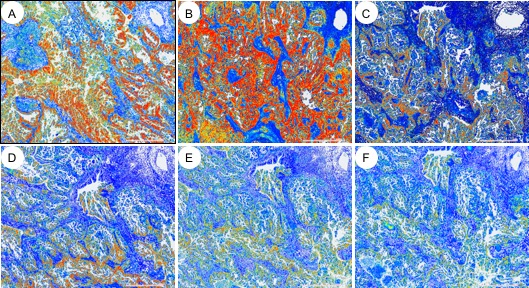

Supplement: S8 Fig — (TIF) [file pone.0183023.s009.tif]
